# Rapid and High-Throughput Detection of Highly Pathogenic Bacteria by Ibis PLEX-ID Technology

**DOI:** 10.1371/journal.pone.0039928

**Published:** 2012-06-29

**Authors:** Daniela Jacob, Uschi Sauer, Roberta Housley, Cicely Washington, Kristin Sannes-Lowery, David J. Ecker, Rangarajan Sampath, Roland Grunow

**Affiliations:** 1 Robert Koch-Institut, Berlin, Germany; 2 Ibis Biosciences, Inc., an Abbott Company, Carlsbad, California, United States of America; 3 Athogen, Inc., Irvine, California, United States of America; Naval Research Laboratory, United States of America

## Abstract

In this manuscript, we describe the identification of highly pathogenic bacteria using an assay coupling biothreat group-specific PCR with electrospray ionization mass spectrometry (PCR/ESI-MS) run on an Ibis PLEX-ID high-throughput platform. The biothreat cluster assay identifies most of the potential bioterrorism-relevant microorganisms including *Bacillus anthracis, Francisella tularensis, Yersinia pestis, Burkholderia mallei* and *pseudomallei, Brucella* species, and *Coxiella burnetii.* DNA from 45 different reference materials with different formulations and different concentrations were chosen and sent to a service screening laboratory that uses the PCR/ESI-MS platform to provide a microbial identification service. The standard reference materials were produced out of a repository built up in the framework of the EU funded project “Establishment of Quality Assurances for Detection of Highly Pathogenic Bacteria of Potential Bioterrorism Risk” (EQADeBa). All samples were correctly identified at least to the genus level.

## Introduction

Highly pathogenic bacteria can often cause infectious zoonotic diseases and even large outbreaks in the human and animal population. A number of these agents have the potential for, or have been used, in bioterrorist attacks [1]–[3]. The intentional release of infectious agents can result in serious public health consequences as shown by the anthrax episodes in the USA in 2001. Rapid and reliable laboratory detection and confirmation strategies for potential bioterrorism agents contribute to reducing the health risk and improving emergency response and public health control initiatives [4].

In case of deliberately or naturally occurring pathogens, the agent must be identified as rapidly as possible to allow measures to be taken to prevent spread and to ensure proper treatment of casualties. In addition, unknown materials accompanied by declarations threatening individual persons, organizations, or events must be analyzed rapidly. In both these scenarios, the assay used must be able to identify a broad panel of potential threat microorganisms, possibly in a background matrix that is contaminated with non-pathogenic bacteria or viruses in order to exclude or confirm the presence of a biological threat.

A number of bacterial pathogens were classified by CDC (Centers for Disease Control and Prevention) as category A and B comprising the highest concern for use in bioterrorist attacks [2], [5]. The categories include bacterial agents such as *Bacillus anthracis, Francisella tularensis, Yersinia pestis, Burkholderia mallei* and *pseudomallei, Brucella* species, and *Coxiella burnetii*. These microorganisms also cause naturally occurring diseases in animals and humans. In most European countries, the natural prevalence of these pathogens is low, although, from time to time some of the pathogens, e.g. *F. tularensis, Brucella* spp., or *C. burnetii* cause outbreaks in animal and human populations. Low disease prevalence and low diagnostic demands are probably the main reasons why existing diagnostic and detection assays are almost not commercially available for this group of zoonotic diseases. Existing diagnostic and detection assays for highly pathogenic bacteria are primarily based on classical microbiological and on molecular and immunological methods, mainly developed as in-house assays. Some multiplex assays have been designed to simplify and shorten the period of identification for pathogens responsible for infectious diseases. However, a highly sensitive yet broadly inclusive approach for the identification of all bacteria under suspicion is not yet available. In addition, due to the lack of reference samples for clinical as well as environmental samples, it is often difficult to validate and determine the accuracy of broad-spectrum assays.

An interesting approach for a complete identification of a large number of microorganisms is the Ibis PLEX-ID technology offered by Abbott [6]–[8]. This technology is based on polymerase chain reaction/electrospray ionization-mass spectrometry (PCR/ESI-MS). A number of primer pairs are used to amplify nucleic acid markers from a wide variety of organisms, followed by an analysis of PCR amplicons using mass spectrometry. The analysis of precise masses of these amplicons allows the calculation of the base compositions of the PCR products; these base compositions are then compared to a database for identification of the pathogen or pathogens. This platform provides a broad infectious disease detection capability and has been used for the detection of bacteria and viruses from a variety of different sources [7], [9]–[12]. In the accompanying manuscript, Sampath et al. [21] describe an approach for the identification of most relevant biothreat agent groups, the so called “biothreat cluster assay”, for the PLEX-ID that uses 36 multiplexed primer pairs for detection of biothreat agents. The biothreat cluster assay was validated by analysis of a broad collection of biothreat organisms and near neighbors prepared by spiking biothreat nucleic acids into nucleic acids extracted from filtered environmental air. The biothreat assay detects 28 NIAID (National Institute of Allergy and Infectious Diseases) priority pathogens [13] and 18 HHS/USDA (United States Department of Health and Human Services/United States Department of Agriculture) select agents [14].

In the framework of the European Union (EU) funded project “Establishment of Quality Assurances for Detection of Highly Pathogenic Bacteria” (EQADeBa) we assembled a repository of the bacterial pathogens mentioned above which we then used to produce standardized reference material. The EQADeBa project provides the infrastructure and the design, the organization, and the management of an external quality assurance (EQA) scheme for potential bioterrorism agents (European Commission, EAHC – Agreement n° - 2007 204). The project includes designing and implementing practical proficiency tests for the detection of bacterial agents of CDC category A and B. Standardized reference materials have been prepared in different formulations, sometimes as mixed cultures and in different concentrations for these exercises. We used material from two proficiency tests, from which 45 different samples were chosen after DNA-preparation for analysis using the biothreat cluster assay and the PLEX-ID technology. All samples were correctly identified as described below.

## Materials and Methods

### Bacterial Cultivation

The bacterial strains used are listed in Table 1 and Table 2. Strains from the EQADeBa repository were plated on blood agar plates with the exception of *Francisella* sp. which was plated on cysteine heart agar (QUELAB, Quebec, Canada). The identity of all used strains was verified by appropriate microbiological and molecular methods. Strains were cultivated under appropriate biosafety level (BSL) 2 or 3 standards. After sterility testing, inactivated suspension or DNA produced under BSL3 conditions were handled under BSL2 conditions. Inactivated cells from *C. burnetii* were kindly provided by Georg Baljer (Justus-Liebig-Universität, Gießen, Germany).

**Table 1:** “DNA samples” for the bacterial strains with DNA concentrations and estimated genome equivalents used, as well as results given by the PLEX-ID technology with genomes per well and Q-score.

Code of “DNA samples”	Decoding: Organism/Strain	Mean value of DNAconcentration [ng/µl]	Genomeequivalents [GE/µl]	Organism detected with PLEX-ID	Level(Genomes/Well)	Q-Score
DNA-1	*E. coli*/A002-43 Le10	9,23	1,68×10^6^	*E. coli* not H7/NoBZ present	1339	1.00
DNA-2	*F. tularensis* subsp. *tularensis/*Ft 12	12,30	5,93×10^6^	*F. tularensis* *	1492	1.00
DNA-3	*B. anthracis/*Jena DU III-7	11,63	1,99×10^6^	*B. anthracis* *	1368	1.00
DNA-4	*B. thailandensis*/DSM 13276	9,65	1,31×10^6^	*B. thailandensis* *	1628	1.00
DNA-5	*Y. pestis*/03-1501	12,77	2,53×10^6^	*Y. pestis*	1251	1.00
DNA-6	*C. burnetii*/Nine Mile RSA 493	13,10	5,98×10^6^	*C. burnetii* *	456	1.00
DNA-7	*F. philomiragia/*DSM 7535	18,67	8,33×10^6^	*F. philomiragia* *	742	0.97
DNA-8	*B. melitensis*/16M	12,50	3,47×10^6^	*B. melitensis* *	1344	1.00
DNA-9	*B. thurigiensis*/DSM 350	24,27	4,22×10^6^	*B. thuringiensis/cereus* *	1303	0.98
DNA-10	*B. pseudomallei*/HO 3460-0149	10,05	1,27×10^6^	*B. pseudomallei/* *xenovorans* *	1038	1.00
DNA-11	*F. tularensis* subsp. *holarctica*/ATCC29684	19,73	9,50×10^6^	*F. tularensis* *	1459	1.00
DNA-12	*B. abortus/*544	10,85	3,02×10^6^	*B. melitensis* *	1308	1.00
DNA-13	*B. mallei/*ATCC 23344	8,30	1,30×10^6^	*B. mallei/pseudomallei* *	1173	1.00
DNA-14	*Y. pseudotuberculosis*/A002-49 Le9	21,03	4,08×10^6^	*Y. pseudotuberculosis*	1055	1.00
DNA-15	*B. cereus*/DSM 31	9,05	1,53×10^6^	*B. thuringiensis/cereus* *	1345	0.98

* *E. coli* was also found in these samples with a Q-score of 1.00.

**Table 2:** “Inactivated Bacterial samples” in different matrices used, as well as results given by the PLEX-ID technology with genomes per well and Q-score.

Code of “Inactivated Bacteria samples”	Decoding: Organisms/Strains	Matrix	Estimated cellsper ml (countingchamber)	Organism detectedwith PLEX-ID	Level(Genomes/Well)	Q-Score
Iso -1	*Y. pestis*/03-1506	PBS	3×10^8^	*Y. pestis*	1140	1.00
Iso-2	*B. anthracis*/UD III-7	PBS	4×10^6^	*B. anthracis* *	1390	1.00
Iso-3	*F. tularensis* subsp. *holarctica*/ATCC29684	PBS	2×10^9^	*F. tularensis* *	1492	1.00
Iso-4	*B. thailandensis/*DSM 13276	PBS	3×10^8^	*B. thailandensis* *	924	1.00
Iso-5	Negative control	PBS	0	*F. tularensis* *	288	1.00
Tiss-1	*F. tularensis* subsp. *holarctica*/ATCC 29684	mouse hybridoma cells	2×10^9^	*F. tularensis* *	1490	1.00
Tiss-2	*B. thailandensis/*DSM 13276	mouse hybridoma cells	3×10^8^	*B. thailandensis* *	865	1.00
Tiss-3	Negative control	mouse hybridoma cells	0	*E. coli*	1114	1.00
Tiss-4	*B. anthracis*/UD III-7	mouse hybridoma cells	4×10^6^	*B. anthracis* *	1303	1.00
Tiss-5	*Y. pestis*/03-1506	mouse hybridoma cells	3×10^8^	*Y. pestis*	1334	1.00
Env-1	*B. anthracis/*UD III-7	river water	4×10^6^	*B. anthracis* *	1828	1.00
Env-2	Negative control	river water	0	*E. coli*	1596	1.00
Env-3	*Y. pestis/*03-1506	river water	3×10^8^	*Y. pestis*	1179	1.00
Env-4	*B. thailandensis*/DSM 13276	river water	3×10^8^	*B. thailandensis* *	1095	1.00
Env-5	*F. tularensis* subsp. *holarctica*/ATCC29684	river water	2×10^9^	*F. tularensis* *	1492	1.00
Env-6	Negative control	lake water	0	*B. vietnamensis,* *E. coli*	2171378	1.001.00
Env-7	*B. pseudomallei*/HO 3460-0149	lake water	1×10^8^	*B. pseudomallei/xenovorans* *	927	1.00
Env-8	*B. pseudomallei*/HO 3460-0149	lake water	1×10^6^	*B. pseudomallei/xenovorans*	517	1.00
Env-9	*Y. pestis*/03-1506	lake water	1×10^8^	*Y. pestis*	3615	1.00
Env-10	*Y. pestis*/03-1506	lake water	1×10^6^	*Y. pestis*	2847	1.00
Food-1	*B. anthracis*/UD III-7	milk	1×10^5^	*B. anthracis* *C burnetii* *	12231870	1.000.98
Food-2	*B. melitensis*/16M	milk	1×10^9^	*B. melitensis* *C. burnetii* *	1482754	1.000.98
Food-3	*B. anthracis*/UD III-7	milk	1×10^6^	*B. anthracis* *C. burnetii* *	128338	1.001.00
Food-4	Negative control	milk	0	*E. coli* *C. burnetii* *C. perfringens*	24132555105	1.000.980.93
Food-5	*B. melitensis*/16M	milk	1×10^7^	*C. burnetii* *B. melitensis* *	1160165	0.980.88
Clin-1	*F. tularensis* subsp. *holarctica*/ATCC 29684	fetal calf serum	1×10^9^	*F. tularensis* *	1494	1.00
Clin-2	*F. tularensis* subsp. *holarctica*/ATCC 29684	fetal calf serum	1×10^6^	*F. tularensis*	1271	1.00
Clin-3	*B. mallei*/ATCC 23344	fetal calf serum	1×10^6^	*B. mallei/pseudomallei* *	413	0.97
Clin-4	*B. mallei*/ATCC 23344	fetal calf serum	1×10^8^	*B. mallei/pseudomallei* *	1021	1.00
Clin-5	Negative Control	fetal calf serum	0	*E. coli*	756	1.00

* *E. coli* was also found in these samples with a Q-score of 1.00.

### “DNA Samples”

15 DNA samples were directly prepared from bacterial cultures and used as reference material (Table 1). Total DNA was purified according to a modified protocol for Gram-negative bacteria using the QIAGEN DNeasy Blood and Tissue kit (Hilden, Germany). Rather than harvest cells from broth media, bacteria from agar plate were inoculated directly into 180 µl ATL buffer. After adding 20 µl proteinase K, we incubated 90 minutes at 56°C followed by adding 200 µl AL buffer for 10 minutes at 70°C. To avoid cross contamination we mixed all samples only by pipetting and added extra centrifugation steps between heat incubation steps. After at least one week of sterility testing, DNA concentrations were measured using the nanodrop 8000 (peqlab Biotechnologie GmbH, Erlangen, Germany). Dilutions of DNA were carried out to 10^6^ copies/µl using AE buffer. The mean values of three DNAs of each bacterium were used to estimate the genome equivalents. Each bacterial DNA was analyzed by real-time PCR to ensure that no cross-contamination had occurred.

### Inactivation of Bacterial Samples

To prepare reference materials, each bacterial species was inoculated into PBS to a density of approx. OD_600_ = 1.0 directly from colonies of agar culture media. To estimate the colony forming units (cfu) of each suspension, dilutions were plated onto blood agar plates (or cysteine heart agar plates for *F. tularensis*). Each sample was tested in duplicate. Bacterial suspensions were inactivated by heating at 60°C for at least 22 h. The exception was *B. anthracis* which was treated by 1% peracetic acid for 30 min with subsequent washing. All inactivated suspensions were checked for sterility by one tenth of each volume over 14 days. After inactivation, cell numbers in each suspension were determined using a counting chamber and real-time PCR. Suspensions of all inactivated samples were analyzed for cross contamination with other target bacteria by real-time PCR and by immunological methods (direct immune fluorescence and ELISA).

### Preparation of “Inactivated Bacteria Samples”

Six different matrices were chosen to spike with the different inactivated bacteria (Table 2): 1) PBS as a substitute for isolate samples (Iso), 2) inactivated mouse hybridoma cells (Ag8) with a concentration of 2×10^6 ^/ml as a surrogate for the tissue samples (Tiss), 3) autoclaved and sterile filtrated natural water from river “Spree” (Env-1 through Env-5), 4) autoclaved water from lake “Plötzensee” as matrix for the environmental samples (Env-6 through Env-10), 5) a reduced-fat, pasteurized and homogenized milk served as a substitute for the food samples (Food), and 6) fetal bovine serum from Invitrogen/Gibco (lot# 41A1268K) was used as surrogate for the clinical samples (Clin). Each bacterial suspension was diluted 1∶10 with the corresponding matrix. Each set of vials for the environmental samples Env-6 to Env-10 and for the food as well as for the clinical samples contained two different target bacteria with two different concentrations. Additionally, each matrix was included as a negative control (Table 2).

### Preparation of DNA from “Inactivated Bacteria Samples” and PLEX-ID Analyses

DNA was isolated from “Inactivated Bacteria samples” using the QIAGEN DNeasy Blood and Tissue kit according to the protocol for Gram-negative bacteria. A total of 45 samples, 30 samples with approx. 100 µl DNA out of the “Inactivated Bacteria samples” and the 15 “DNA samples” were sent for analyses to AthoGen [15], a service screening laboratory that uses the PCR/ESI-MS platform to provide a microbial identification service. PCR was performed in a 50 µL reaction volume containing 5 µL nucleic acid extract in a reaction mix as previously described [9]. One-step real time (RT)-PCR was performed in wells that targeted viral detection. Reverse transcription PCR cycling conditions were used for both RT-PCR and PCR reactions according to previously described methods [7], [10]–[12]. Samples and reaction components were aliquoted into PLEX-ID biothreat cluster assay (Cat. No. 03N35-63) plates using a Janus Automated Workstation. One 96 well plate allows to analyze six samples in parallel, means 16 wells per sample. Thermocycling was carried out on an Eppendorf Mastercycler. After thermocycling, plates were stored at –40°C until the samples could be analyzed by ESI/MS. After PCR amplification, 30 µL aliquots of each PCR reaction were desalted and analyzed by mass spectrometry as previously described [6], [10].

### Data Analysis and Results Reporting

Data analysis and results reporting was performed as described in Sampath et. al. [21]. Briefly, a customized reporting rule set was designed that allowed rapid and accurate detection of the biothreat targets. The biothreat assay report provides data obtained using 36 primer pairs. Each of the threat clusters is treated independently and the results are reported for each cluster separately. Mixed detections of two or more threats or a threat with an unrelated near neighbor in another group are also reported. The system is also capable of reporting organism/strain level matches based on the genomic sequence data in the PLEX-ID database. This is provided as a research utility tool in a separate analysis workstation. Additional details for each of the matches, including the detected base compositions, Q-scores and levels, are available using this report and are described in the accompanying manuscript.

## Results

The PLEX-ID biothreat cluster assay (Ibis Biosciences, Carlsbad, CA) described in the accompanying manuscript by Sampath et al. [21] provides the capacity to analyze samples for targets as *B. anthracis*, *Y. pestis*, *F. tularensis*, *Vibrio cholerae*, *C. burnetii*, as well as *Rickettsia*, and Orthopoxvirus or Filovirus. The 36 primer pairs used in the assay are multiplexed such that 16 wells on a 96-well plate are required per sample; six samples can be analyzed in parallel on a single plate. The PLEX-ID assay system correctly identified, with one exception, all of the 45 unknown samples targeting highly pathogenic bacteria, using DNA template from these organisms (Table 1, DNA-12). In one case, *Brucella abortus* was recognized as *Brucella melitensis*. At the same time, samples containing *B. melitensis* were analyzed correctly (Table 1: DNA-8; Table 2: Food-2, Food-5).

In analyses of “DNA samples” and spiked “Inactivated Bacteria samples” containing *Burkholderia*, *B. mallei* (Table 1: DNA-13; Table 2: Clin-3, Clin-4) could not be discriminated from *B. pseudomallei.* Further, signatures for some isolates of *B. pseudomallei* (Table 1: DNA-10; Table 2: Env-7, Env-8) could not be differentiated from *Burkholderia xenovorans* (Table 1: DNA-10; Table 2: Env-7, Env-8). This might be a problem for risk assessment because *B. pseudomallei* is classified in risk group 3, whereas *B. xenovorans* is classified in risk group 1 and is not pathogenic for humans or animals. This limitation has been described by Sampath et. al. Interestingly, samples containing *Burkholderia thailandensis* (risk group 1), the nearest relative of *B. pseudomallei*, were clearly identified (Table 1: DNA-4; Table 2: Iso-4, Tiss-2, Env-4).

Different samples containing *F. tularensis* were always identified correctly at the species level (Table 1: DNA-2, DNA-11; Table 2: Iso-3, Tiss-1; Env-5, Clin-1, Clin-2), but there is no differentiation between the subspecies in the species summary reporting. Since strain differentiation would be of interest because subsp. *tularensis*, subsp. *holarctica*, and subsp. *mediasiatica* cause tularemia in humans, whereas subsp. *novicida* shows a low virulence and causes very rarely disease in immune-compromised individuals. In this case, a secondary data evaluation would be offered by the PLEX-ID platform using appropriate software that contains detailed subspecies information, and researchers should use this offer if a *Francisella* subspecies is identified. This approach was not applied in our study. A sample comprising a distinct *Francisella* species, *Francisella philomiragia,* was correctly identified (Table 1: DNA-7).

Several samples analyzed contained identical bacteria with different quantities. There was reasonable agreement between quantitative analysis of bacterial numbers obtained by the counting of bacteria in counting cell chambers and the “Genomes per well” calculated by PLEX-ID technology based on a calibrant in the assay wells for *B. pseudomallei* (Env-7 and Env-8), *Y. pestis* (Env-9 and Env-10), *F. tularensis* (Clin-1 and Clin-2), *B. melitensis* (Food-2 and Food-5), and *B. mallei* (Clin-3 and Clin-4). No significant difference in quantities of *B. anthracis* present in samples Food-1 and Food-3 was observed using the PLEX-ID technology although previous counting in the sample preparation process revealed a 10-fold difference.

The closely related species *Y. pestis* and *Yersinia pseudotuberculosis* as well as *B. anthracis* and other *Bacillus cereus*-group strains were correctly identified using the PLEX-ID biothreat cluster assay. *Bacillus thuringiensis* and *B. cereus* were not differentiated (Table 1: DNA-9, DNA-15), consistent with the description in Sampath et. al.

There were observed some anomalous results, yelding in false positive and false negative data or indications of potential cross-contaminations in some of the assays. In one negative control (Table 2: Iso-5) a low genome number of *F. tularensis* was detected, partly caused by the very high concentrations of the bacteria in some samples. The source of the contamination problem remains to be determined. The generation of the reference material and the DNA extraction process that were performed within the laboratory at the Robert Koch-Institut was shown not to be the cause of the contamination. The unexpected identification of *Clostridium perfringens* in sample Food-4 (negative control milk) should be clarified. The detection of *Burkholderia vietnamensis* in sample Env-6 comprising natural lake water could be explained by a cross reactivity or presence of naturally occurring DNA.

In addition to specific targets, *C. burnetii* DNA could be detected in the commercial milk matrix. The presence of this bacterium was confirmed by the External Quality Assurances Exercises (EQAEs) in the framework of the EQADeBa project.

In most of the DNA samples (Table 1) and several other samples (Table 2) traces of *Escherichia coli* DNA were detected. It is not possible to evaluate retrospectively, whether this contamination was due to the amplification or the measurement process.

In conclusion agents present in the samples can be identified with a high level of confidence.

### Discussion

The biothreat assay performed using the PLEX-ID technology can be used to detect a broad collection of biothreat organisms and near neighbors as described in the accompanying manuscript. This assay uses 36 pairs of multiplexed primers to amplify bacterial or viral DNA present in environmental or clinical samples. After analysis of the amplicons by mass spectrometry, base counts of the amplicons are determined. By comparison with the more than 850000 entries in the PLEX-ID database, biothreat agents present in the sample can be identified. There are other multiplex PCR or MALDI-TOF mass spectrometry based methods for analysis of infectious agents [16]–[20]; however, these methods are less broad than the PLEX-ID technology. The PLEX-ID approach provides the possibility for a simultaneous differentiation of multiple pathogens resulting in quantitative data given by “Genomes per well” and the probability of correct identification calculated by the “Q-Score”.

We used 45 samples from External Quality Assurances Exercises (EQAEs) performed in the framework of the above-mentioned EU project on quality assurance of the diagnosis of highly pathogenic bacteria (EQADeBa) to evaluate the ability of the PLEX-ID to correctly identify pathogens within this biothreat cluster. In addition, we assessed the capability of the assay system to differentiate between the more highly pathogenic species from congeners that were either low or apathogenic.

In the current configuration of the instrument, DNA was extracted in a separate step. An integrated DNA extraction step could be helpful.

In general, the biothreat assay run on the PLEX-ID technology had an identification rate of 100% for the highly pathogenic target bacteria at the genus level for the 45 reference samples. With exception of *Burkholderia* and in one case *Brucella*, all other target bacteria could also been discriminated at the species level. In addition to the correct and rapid identification of the target bacterial DNA we have seen several unspecific reactions. Most striking was the contamination with *E. coli* DNA. In parallel cultures of the primary samples, *E. coli* was not found except sample DNA-1 which was prepared from *E. coli* cultures and was used as the source for the DNA-control samples in the first set of experiments (Table 1, data not shown). Thus, an intrinsic contamination during the amplification or measurement process of the sample set must be excluded. Most unexpected results were observed in the negative control samples, where for one negative control a low genome number of *F. tularensis* was detected (Table 2: Iso-5) and is assumed to be the result of cross-contamination.

The ability to differentiate between *B. pseudomallei* and *B. xerovorans* would be most useful as related to potential medical treatment decisions to be made or for the purposes of microbial forensic analysis. Mainly for forensic questions, but also for threat estimation the differentiation of *B. mallei* and *pseudomallei* as well as of *F. tularensis* subspecies would be of interest. In addition, the species *F. tularensis* contains subspecies *tularensis*, *holarctica*, and *mediasiatica*, pathogenic to humans, and subspecies *novicida*, not pathogenic to humans, which are also of interest to be identified. Currently these are distinguished by the assay and details of speciation are available only in a secondary report rather than the primary sample report.

The PLEX-ID *Genomes per well* data reflects the same general trend of quantification as the reference method; however, the magnitudes were quite different. The PLEX-ID assays are only semi-quantitative and *Genomes per well* reflects a relative measure of the abundance as compared to the internal standard [6]. The normal range for reporting these levels is between 0.1X and 10X the levels of internal controls in the assay, which in the case of the PLEX-ID biothreat assay represents a working range of ∼10 GE/well to 1000 GE/well. Above 1000 GE/well, the target organism levels far exceed the calibrant, thus, the reported level only serves to indicate high target concentrations. This would explain that quantitative differences in some samples were not detected.

The PLEX-ID technology is a high-throughput technology that allows rapid analysis of the DNA content of environmental and clinical samples. Here we evaluated 45 samples of genomic DNA in various matrices. The high specificity and high sensitivity of the PLEX-ID biothreat assay reported in the accompanying manuscript by Sampath et al. [21] was confirmed by our analysis of these bacterial samples. An advantage of this assay is that it is semi-generic; a wide range of bacterial agents and their near-neighbours are identified without need for assumptions about the pathogens present. If more than one pathogen is present in a sample, all are identified simultaneously. The results are scored for a probability, which allows the interpretation of uncertain results in the context of further information. The PLEX-ID instrument is useful for the application in central service facilities with a high-throughput of samples.
